# Zinc about it – zinc and calf immunity

**DOI:** 10.3389/fimmu.2024.1387950

**Published:** 2024-05-10

**Authors:** Kathryn W. Kesler, Angel Abuelo

**Affiliations:** Department of Large Animal Clinical Sciences, College of Veterinary Medicine, Michigan State University, East Lansing, MI, United States

**Keywords:** micronutrient, trace mineral, bovine, health, preweaning disease

## Abstract

Micronutrients, such as vitamins and trace minerals, are critical for supporting growth, performance, health and maintaining redox balance. Zinc (Zn), an essential micronutrient, aids the functioning of innate and adaptive immune cells. This scoping review aims to assemble and evaluate the evidence available for the role of Zn within calf immunity. Relevant literature was identified within Web of Science, PubMed, and CABI using search terms specific to the major innate and adaptive immune cell populations. There was no evidence that Zn supplementation altered neutrophil, natural killer cell, or T-cell functions. However, there was limited evidence to support Zn supplementation with reduced monocyte numbers, but there was no evidence to associate the monocytopenia with improvements in monocyte function. There is moderate evidence to suggest that Zn supplementation was beneficial for maintaining epithelial barriers of integumental and mucosal surfaces. The evidence supports supplementation above the current industry recommendations for improving immunoglobulin (Ig) production, with the strongest results being observed for IgG and IgM. Moreover, Zn supplementation was associated with reduced proinflammatory cytokine production, which may reduce inflammation-associated hypophagia and warrants further investigation. Furthermore, Zn reduced the duration of clinical signs in animals facing respiratory disease and diarrhea. However, consensus is needed about the optimal dose, route, and Zn formulation most appropriate for supporting immunity. In conclusion, while the literature supports that Zn could enhance calf immunity, there is insufficient evidence to adequately determine the extent to which Zn impacts innate immune cell and T-cell functions. Determination of the immune cell functions susceptible to modification by Zn supplementation is an important knowledge gap for enhancing the understanding of Zn and calf immunity.

## 1 Introduction

Micronutrients comprise a small percentage of the diet by volume; however, they are pivotal in optimizing growth, performance, immune function, and redox balance (1). Dairy calves experience oxidative stress (OS), the cell damage that results from redox imbalance (2), throughout the preweaning period (3). This OS impairs immune function (4). Recent research has shown that antioxidant supplementation supports a more robust immune response in dairy calves (5, 6).

Zinc is the most abundant metal found in humans and acts as an essential cofactor for numerous enzymes, with approximately 10% of the human proteome being able to bind Zn (7). Severe Zn deficiency is detrimental to immune function in various species, including cattle (8–10). Consequently, diets are routinely formulated to prevent Zn deficiency (11). In humans and adult cattle, there is evidence to support the role of Zn in maintaining redox balance and supporting immune function during critical periods of physiologic stress (12, 13). However, more research is needed to support Zn supplementation above recommendations in dairy calves. This scoping review aims to assemble and evaluate the evidence available for the effect of Zn on calf immunity. This will be followed by a more specific discussion about how these changes in immune function translate into calf health improvements.

## 2 Scoping review methodology

**Figure 1:**
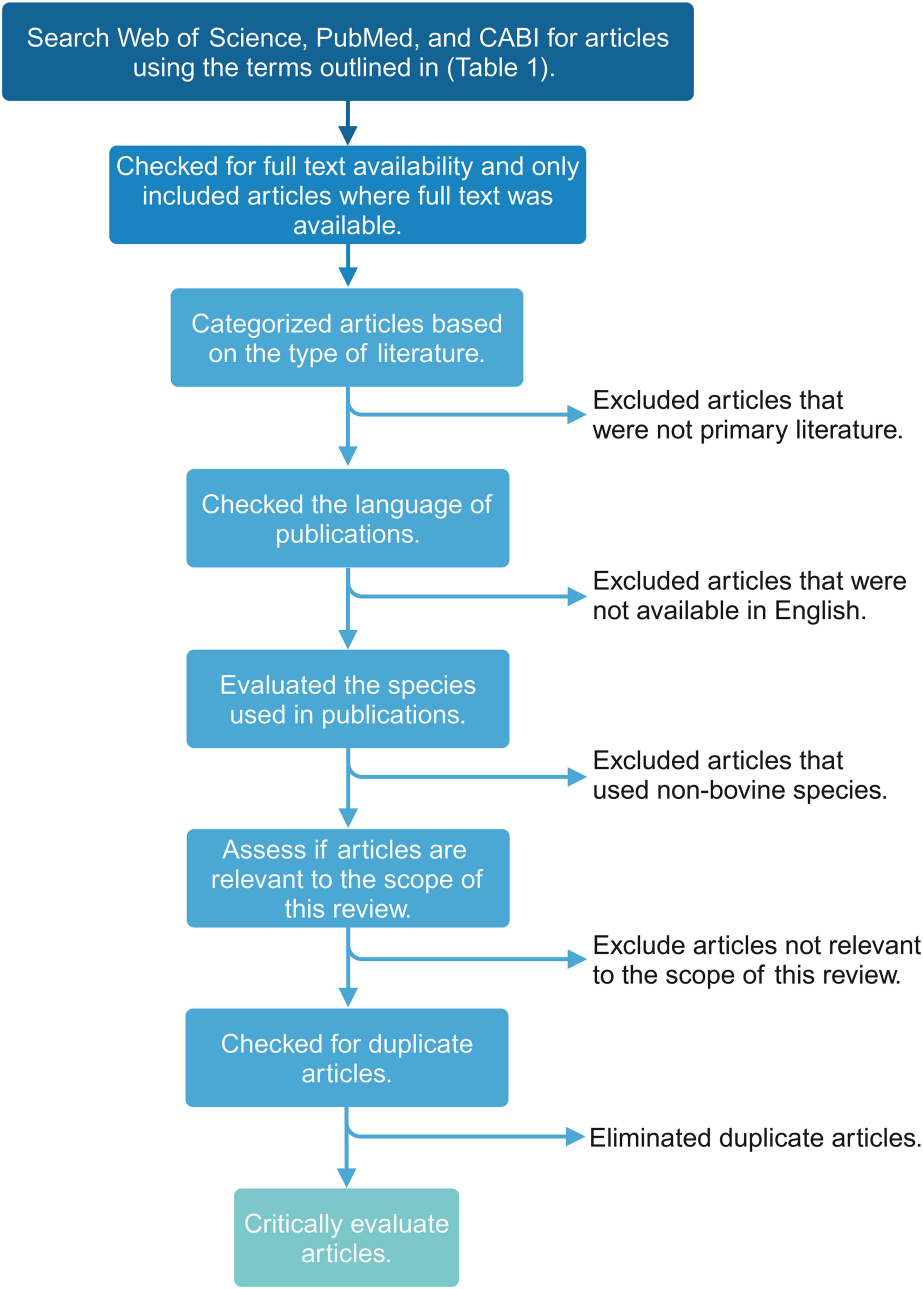
Flowchart visualizing the scoping review methodology and workflow for applying inclusion and exclusion criteria to the search results. This figure was created using BioRender.com.

**Figure 2:**
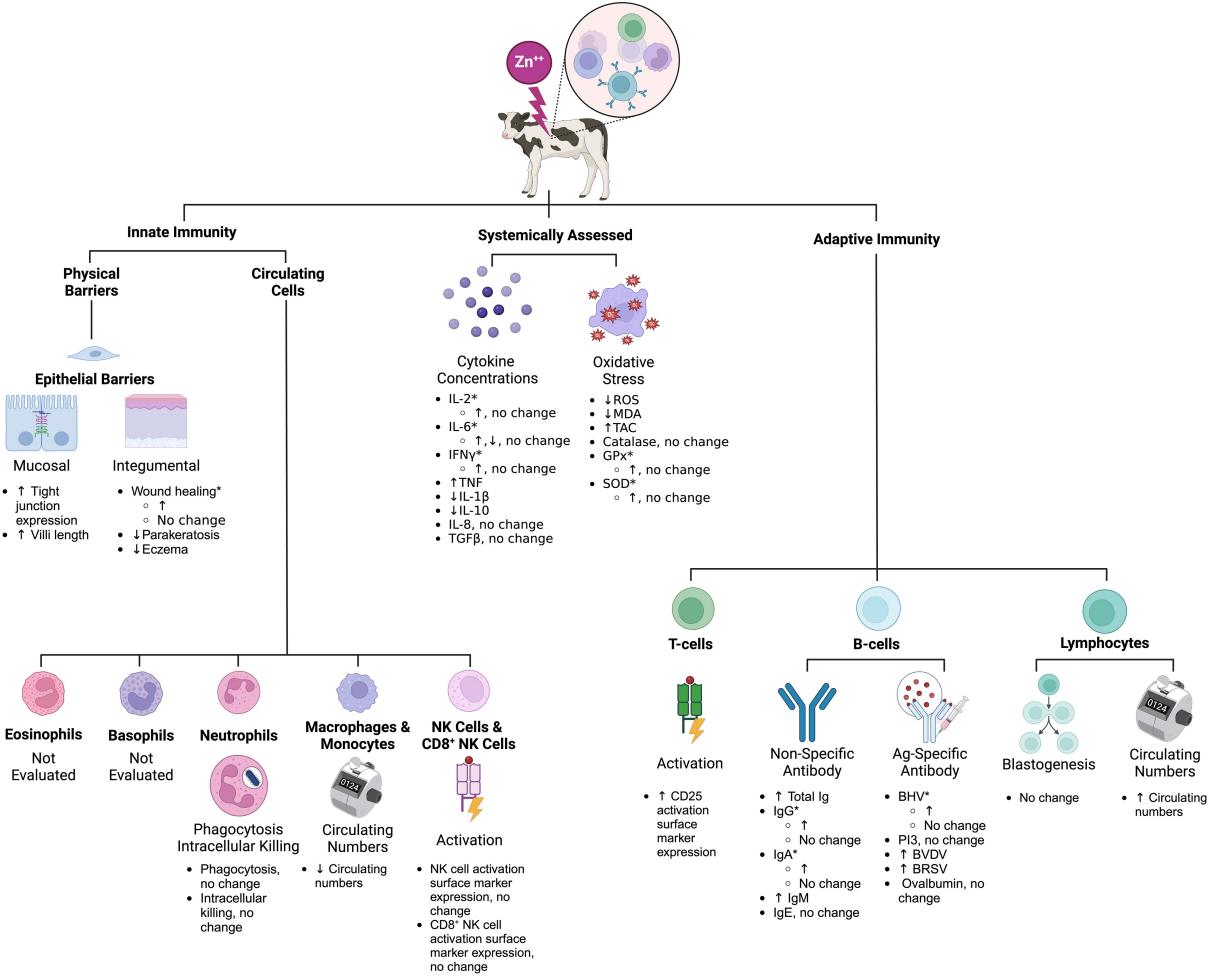
Schematic summary of the effects of Zn on calf immunity based on the scoping review search. *Inconsistent results across studies (see **Tables 4**–**10** for specific study details). Zn, zinc; BHV, bovine herpes virus; BVDV, bovine viral diarrhea virus; BRSV, bovine respiratory syncytial virus; PI3, parainfluenza 3 virus; Ig, immunoglobulin; IL, interleukin; CD, cluster of differentiation; MDA, malondialdehyde; TAC, total antioxidant capacity; GPx, glutathione peroxidase; SOD, superoxide dismutase. This figure was created using BioRender.com.

Unique terms for the major cell types within the innate and adaptive immune systems were selected to ensure the search was comprehensive. The terms described in **Table 1** were used to identify relevant Web of Science, PubMed, and CABI literature. Subsequently, inclusion and exclusion criteria (**Table 2**) were applied, and duplicates were removed to identify the articles relevant to each search category (**Figure 1**, **Table 3**). Each cell type was evaluated and reported independently throughout the article for organizational purposes. Although we considered T- and B-cells separate search categories, several articles evaluated lymphocytes altogether. Therefore, T-cell- and B-cell-specific outcomes were described in unique sections, and non-cell-type-specific outcomes were described in a general lymphocyte section. A schematic summary of the findings is also included (**Figure 2**).

**Table 1:** Search categories and terms for the specific immune cell sections.

Search Category	Search Terms
Neutrophils	(Zinc OR Zn) AND (bovine OR calves OR calf OR cattle) AND (neutrophil)
Monocytes/Macrophages	(Zinc OR Zn) AND (bovine OR calves OR calf OR cattle) AND (monocyte OR macrophage)
Other Innate Immune Cells	(Zinc OR Zn) AND (bovine OR calves OR calf OR cattle) AND (mast OR eosinophil OR basophil OR dendritic OR natural killer OR NK)
Barrier Integrity	(Zinc OR Zn) AND (bovine OR calves OR calf OR cattle) AND (skin OR epithelial OR endothelial OR wound OR barrier)
T-cells	(Zinc OR Zn) AND (bovine OR calves OR calf OR cattle) AND (Lympho* OR T cell OR Tcell OR T lympho* OR CD8 OR CD 8 OR CD4 OR CD 4 OR CD3 OR CD 3)
B-cells	(Zinc OR Zn) AND (bovine OR calves OR calf OR cattle) AND (Lympho* OR B cell OR Bcell OR B lympho* OR humoral OR antibody OR immunoglobulin OR titer)

**Table 2:** Inclusion and exclusion criteria applied to the search results for the neutrophil, monocyte/macrophage, other innate immune cells, T-cell, and B-cell sections.

Category	Criteria	Examples
Inclusion	Only full-text journal articles	Included only full-text journal articles (e.g., not conference proceedings/meeting abstracts, reports, book chapters, or databases)
Exclusion	Not primary literature	Literature reviews
Exclusion	Non-bovine	Articles utilizing zinc with a non-bovine species such as sheep, swine, or humans.
Exclusion	Non-English	Articles only available in a non-English language.
Exclusion	Not relevant to the scope of this review	Articles that did not evaluate immune outcomes, used mixed trace mineral sources, did not evaluate the age of interest, or did not utilize zinc.

**Table 3:** Results returned from each search and the application of the inclusion and exclusion criteria resulting in the unique number of articles critically evaluated for each section.

Database	Total articles retrieved	Including only full-text journal articles	Excluded articles				Total relevant	Total relevant after removing duplicates	References evaluated
			Not primary literature	Non-English articles	Not relevant to the scope of this review	Non-bovine articles			
Neutrophils									
Web of Science	182	171	10	4	155	1	1	1	(14)
PubMed	66	66	3	1	60	1	1		
CABI	82	79	3	1	73	1	1		
Monocytes/macrophages									
Web of Science	93	92	1	31	58	0	2	2	(15, 16)
PubMed	81	81	0	17	61	1	2		
CABI	52	51	0	9	40	1	1		
Other innate immune cells									
Web of Science	39	38	4	0	33	0	1	1	(17)
PubMed	30	30	1	1	26	1	1		
CABI	21	18	0	0	17	1	0		
Barrier integrity									
Web of Science	450	431	20	0	407	1	3	9	(18–26)
PubMed	439	436	5	0	426	2	3		
CABI	445	386	18	36	320	3	9		
T-cells									
Web of Science	856	849	18	14	810	0	7	7	(14–17, 27, 28)
PubMed	383	383	6	7	354	11	5		
CABI	504	456	4	8	429	12	3		
B-cells									
Web of Science	910	893	31	0	839	11	12	12	(14–16, 27–35)
PubMed	436	432	14	1	401	4	11		
CABI	522	474	25	20	407	8	8		

## 3 Zinc and innate immunity

### 3.1 Neutrophils

No change in neutrophil function was reported after feeding low (60 milligram (mg) per kilogram (kg) dry matter (DM)) and medium (150 mg/kg DM) doses of Zn Methionine (Met)-Zn Lysine (Lys) or a high (300 mg/kg DM) dose of Zn oxide to 6-week-old dairy heifers (**Table 4**) (14). Neutrophil phagocytosis and intracellular killing were evaluated ex vivo at 0, 2, 4, and 6 weeks of the study (14). It is important to highlight that Zn treatments below 300 mg/kg DM did not increase serum or liver Zn concentrations, making it difficult to interpret a lack of change in neutrophil function at lower doses. Furthermore, there was no reduction in neutrophil phagocytosis or intracellular killing to suggest immuno-suppression, which had been observed with high Zn doses *in vitro* (36). Based on this research, there is insufficient evidence to determine the extent to which Zn supplementation can alter neutrophil function in dairy calves.

**Table 4:** Summarized results of the neutrophil search.

Type of study (N, # groups)	Basal diet Zn	Zn supplement & formulation [Zn dose]	Route	Duration of supplementation	Animals	Outcomes*	Findings	Reference
RCT (N=40, 4)	60 mg/kg	A. 0 mg/kg Zn Met & Zn Lys B. 150 mg/kg Zn Met & Zn Lys [15 mg/kg Zn] C. 300 mg/kg Zn Met & Zn Lys [30 mg/kg Zn] D. 300 mg/kg Zn oxide [168 mg/kg Zn]	PO	6 weeks	6-8 week old Holstein heifer calves	Phagocytosis Intracellular killing	Phagocytosis No difference Intracellular killing No difference	(14)

RCT, randomized controlled trial; Zn, zinc; Met,methionine; Lys, lysine; mg, milligram; kg, kilogram; PO, per oral. *Only stimulations and outcomes relevant to the assessment of neutrophils are listed.

### 3.2 Monocytes/macrophages

Zn supplementation had mixed results on monocyte counts with a tendency to decrease numbers (**Table 5**) (15, 16). However, an assessment of the effect of Zn on monocyte function was not performed in these studies. Zn-supplemented 50-day-old calves had reduced circulating monocyte numbers 40 days after subcutaneous supplementation of Zn edetate (1 mg/kg body weight) or diphenyl diselenide (0.3 mg/kg body weight) combined with Zn edetate (1 mg/kg body weight) (15). In contrast, circulating monocyte counts were increased for 24 hours after being challenged with bovine herpes virus (BHV) and *Mannheimia haemolytica* to cause bovine respiratory disease (BRD) in heifers (272 ± 3.4 kg) treated with Zn sulfate (100 mg/kg DM or 200 mg/kg DM) or combined supplementation of Zn Met – Zn sulfate (80 mg/kg DM - 20 mg/kg DM) for 30 days (16). Heifers who received the combined supplementation tended to have the lowest circulating monocyte values from day -3 to 7 post-challenge. Improved monocyte functionality could explain the reduction in circulating immune cell numbers. However, neither article evaluated monocyte function, making it difficult to appreciate Zn supplementation’s impact on monocytes beyond being associated with reduced circulating numbers. Future research is warranted to investigate the role of Zn supplementation on specific monocyte functions such as phagocytosis, respiratory burst, or activation. Ultimately, there is insufficient evidence to make conclusions about the extent to which Zn supplementation alters monocyte numbers and functionality.

**Table 5:** Summarized results of the monocyte/macrophage search.

Type of study (N, # groups)	Basal diet Zn	Zn supplement & formulation [Zn dose]	Route	Duration of supplementation	Animals	Outcomes*	Findings	Reference
RCT (N=24, 4)	Not reported	A. 0 mg/kg BW Zn edetate & 0 mg/kg Diphenyl diselenide B. 1 mg/kg BW Zn edetate [0.15 mg/kg Zn] C. 0.3 mg/kg BW Diphenyl diselenide [0 mg/kg Zn] D. 1 mg/kg BW Zn edetate & 0.3 mg/kg BW Diphenyl diselenide [0.15 mg/kg Zn]	s.c.	The study lasted 300 days. Supplementation occurred twice on study day 1 and 20.	50 day old male Holstein calves	Circulating numbers on study days 1, 20, and 40.	B, C & D < A, day 40	(15)
RCT (N=32, 3)	Not reported	A. 100 mg/kg DM Zn sulfate [40.5 mg/kg Zn] B. 200 mg/kg DM Zn sulfate [81 mg/kg Zn] C. 80 mg/kg DM Zn Met & 20 mg/kg DM Zn sulfate [24.9 mg/kg Zn]	PO	30 days	255 ± 15 kg beef heifers	Circulating numbers after challenge with BRD pathogens	C < A & B	(16)

RCT, randomized controlled trial; Zn, zinc; mg, milligram; kg, kilogram; Met, methionine; s.c., subcutaneous; PO, per oral; BW, body weight; DM, dry matter; BRD, bovine respiratory pathogens. *Only stimulations and outcomes relevant to the assessment of monocytes/macrophages are listed.

### 3.3 Other innate immune cells

Zinc supplementation (1,000 mg/day) did not alter the expression of activation surface markers (CD2, CD16, CD25, CD44, CD45RO) in natural killer (NK) cells in weaned beef steers (**Table 6**) (17). In contrast, when the same surface markers were assessed in CD8^+^ NK cells, there was increased CD16 expression (**Table 6**) (17). These results indicate that Zn supplementation can alter phenotypic activation markers; however, conclusions were limited by inconsistent expression of activation surface markers (17). Future work validating activation marker expression with functional immune outcomes would help elucidate Zn’s role in NK cell function.

**Table 6:** Summarized results of the other innate immune cell search.

Cell Type	Type of study (N, # groups)	Basal diet Zn	Zn supplement & formulation [Zn dose]	Route	Duration of supplementation	Animals	Outcomes*	Findings	Reference
NK	RCT (N=72, 3)	0 mg/kg	A. 7 g/d organic trace mineral mix [951 mg/d Zn]	PO	42 days	284 ± 25 kg angus crossbred steers	Activation surface marker expression without stimulation (CD2, CD16, CD25, CD44, CD45RO)	No difference	(17)
			B. 7 g/d organic trace mineral mix & 1000 mg/d Zn AA for 14 days then only the organic trace mineral mix [1900, 929 mg/d Zn]		14 days, 28 days				
			C. Inorganic trace mineral mix formulated to match group A. [1095 mg/d Zn]		42 days				
CD8^+^ NK	RCT (N=72, 3)	0 mg/kg	A. 7 g/d organic trace mineral mix [951 mg/d Zn]	PO	42 days	284 ± 25 kg angus crossbred steers	Activation surface marker expression without stimulation (CD2, CD16, CD25, CD44, CD45RO)	CD25 MFI C > A & B, day 42 % CD45RO cells B > A & C, day 0	(17)
			B. 7 g/d organic trace mineral mix & 1000 mg/d Zn AA for 14 days then only the organic trace mineral mix [1900, 929 mg/d Zn]		14 days, 28 days				
			C. Inorganic trace mineral mix formulated to match group A. [1095 mg/d Zn]		42 days				

RCT, randomized controlled trial; Zn, zinc; mg, milligram; kg, kilogram; g, gram; d, day; AA, amino acid complex; PO, per oral; CD, cluster of differentiation; MFI, mean fluorescence intensity; NK, natural killer cells. *Only stimulations and outcomes relevant to the assessment of other innate immune cells are listed.

### 3.4 Barrier integrity

Epithelial surfaces are anatomic barriers that constantly protect from invading pathogens, representing an important but rudimentary component of the innate immune system (37). Many clinical signs associated with hypozincemia represent skin dysfunction, including alopecia, rough coat, dry and scaling skin, and skin lesions (8). These clinical signs respond well to Zn supplementation and are often self-limiting (8, 38). Parakeratosis is a Zn-responsive skin condition in dairy cattle that commonly occurs in young animals, dry cows fed large amounts of calcium, and during Zn deficiency. Regardless of the cause, the lesions resolve with 500 mg/day – 2,000 mg/day Zn sulfate supplementation (18, 19). Moreover, skin lesions associated with tail root eczema respond well to Zn therapy (Zn chloride or Zn oxide) with 800 mg/day resolving clinical signs 3 to 9 days faster than 240 mg/day (20).

Fungal skin infections such as facial eczema caused by the fungal toxin sporidesmin respond well to zinc supplementation. Facial eczema in calves 6-12 months of age, was successfully treated with 60 mg Zn sulfate per 1 liter of water for 28 days (21). Moreover, a Zn oxide bolus protected calves (6-12 months of age) that were exposed to pastures laden with sporidesmin (22). Although there were no clinical signs of facial eczema, Zn-supplemented animals had less liver damage, as evidenced by reduced hepatic enzyme release. Contracture and epithelialization are important skin functions for wound healing. Wound healing of surgically created lesions was faster with Zn oxide supplementation (40 mg/kg DM) when weaned calves were Zn deficient (39); however, when 400 mg/kg DM of Zn sulfate and Zn oxide were supplemented to 6-month-old heifers without Zn deficiency, there was no difference in the rate of wound closure (23). This suggests the existence of a threshold of Zn concentrations for wound healing, with supplementation above that cutoff being of limited value.

Epithelial barriers line mucosal surfaces and prevent pathogen invasion, especially in the gastrointestinal tract. *In vitro*, Zn oxide nanoparticle supplementation (0.8 microgram per milliliter) increased the viability of bovine intestinal epithelial cells (24). Intestinal epithelial cells were harvested from 1-day-old dairy calves; the cells were heat treated to model heat stress *in vitro*. Zn-supplemented cells had increased viability, reduced apoptosis, and elevated tight junction gene expression. Similarly, neonatal dairy calves supplemented with 80 mg/day Zn Met had improved intestinal mucosal barrier as measured by increased ileal villi height and increased gene expression of jejunal tight junctions (25). As discussed later, these improvements could explain Zn’s positive effects in managing diarrhea. In conclusion, there is strong evidence to suggest that Zn supplementation is essential for the epithelial integrity of integumental and mucosal surfaces. Heterogeneity of the dose, route, and formulation of Zn limited comparisons among studies and future research optimizing Zn supplementation is needed.

## 4 Zinc and adaptive immunity

### 4.1 T-cells

The capacity for T-cells to rapidly change from a quiescent to an activated state when the immune system has been stimulated is essential for a robust immune response to vaccines and pathogens alike (40, 41). Zn supplementation only improved CD 25 activation surface marker expression with inorganic supplementation at 42 days in CD8^+^ T-cells. Organic supplementation as an amino complex did not improve any of the studied activation surface markers (CD2, CD16, CD25, CD44, CD45RO) in CD8^+^ T-cells with or without 14 days of additional Zn (1,000 mg/day) in weaned beef steers (**Table 7**) (17). Many subpopulations of T-cells contribute to an immune response; future work evaluating more populations of T cells, such as T helper, T regulatory, and gamma-delta T-cells, would increase our understanding of the impact of Zn on T-cell function.

**Table 7:** Summarized results of the T-cell search.

Type of study (N, # groups)	Basal diet Zn	Zn supplement & formulation [Zn dose]	Route	Duration of supplementation	Animals	Outcomes*	Findings	Reference
RCT (N=72, 3)	0 mg/kg	A. 7 g/d organic trace mineral mix [951 mg/d Zn]	PO	42 days	284 ± 25 kg angus crossbred steers	Activation surface marker expression without stimulation (CD2, CD16, CD25, CD44, CD45RO)	CD25 MFI C > A & B, day 42 % CD45RO cells B > A & C, day 0	(17)
		B. 7 g/d organic trace mineral mix & 1000 mg/d Zn AA for 14 days then only the organic trace mineral mix [1900, 929 mg/d Zn]		14 days, 28 days				
		C. Inorganic trace mineral mix formulated to match group A. [1095 mg/d Zn]		42 days				

RCT, randomized controlled trial; Zn, zinc; mg, milligram; kg, kilogram; g, gram; d, day; AA, amino acid complex; PO, per oral; CD, cluster of differentiation. *Only stimulations and outcomes relevant to the assessment of T-cells are listed.

### 4.2 B-cells

Immunoglobulins are an essential effector function of plasma cells and terminally differentiated B-cells, providing critical protection against pathogens (42). There were inconsistent results in immunoglobulin outcomes with Zn supplementation (**Table 8**). Zinc supplementation increased total immunoglobulin (Ig) in serum and plasma when Zn (30 - 32 mg/kg DM) was fed to preweaning calves (29, 30). Increased IgG concentrations were reported when 32-100 mg/kg DM Zn was supplemented to preweaning dairy calves (27, 29, 31, 32). In contrast, 1-month-old beef calves supplemented with a Zn amino acid complex (40, 80, 120 mg/kg DM) showed no difference in IgG concentrations among the treatments (33). Serum and plasma IgM concentrations increased in preweaning calves when 32-100 mg/kg DM Zn was supplemented (27, 31–33). However, a threshold of Zn concentration seems to be in place as no further improvement was documented in beef calves supplemented with 120 mg/kg DM (33). Zn supplementation (32 mg/kg DM) in the form of nanoparticles increased plasma IgA concentrations (29). However, when Zn was supplemented as Zn oxide (40, 80, 120 mg/kg DM), Zn proteinate (80 mg/kg DM), Zn as an amino acid complex (40, 80, 120 mg/kg DM) or Zn Met (80 mg/kg DM) there were no changes in preweaning calf serum or plasma IgA concentrations (27, 29, 31, 33). It is important to note that IgA is most abundant in external secretions; thus, serum and plasma samples may not be representative of changes in IgA production (44). Zn supplementation did not alter plasma IgE concentrations (29).

**Table 8:** Summarized results of the B-cell search.

Type of study (N, # groups)	Basal diet Zn	Zn supplement & formulation [Zn dose]	Route	Duration of supplementation	Animals	Outcomes*	Findings	Reference
RCT (N=24, 4)	Calf starter: 46.39 mg/kg DM Wheat straw: 4.29 mg/kg DM Berseem fodder: 19.28 mg/kg DM Milk: 3491 g/L	A. 0 mg/kg DM Zn or Cu nanoparticles B. 32 mg/kg Zn nanoparticles [25.6 mg/kg Zn] C. 10mg/kg Cu nanoparticles [0 mg/kg Zn] D. 32 mg/kg Zn nanoparticles & 10mg/kg Cu nanoparticles [25.6 mg/kg Zn]	PO	14 days	1 month old dairy calves	Antibody concentration without stimulation	Total immunoglobulin D > B & C > A IgG D > A & C IgA D > A IgM D > A & B IgE No difference	(29)
RCT (N=20, 2)	Calf starter: 30 mg/kg DM Milk replacer: 40 mg/kg DM	A. 0 mg/kg DM Zn Met B. 30 mg/kg DM Zn Met [6.3 mg/kg Zn]	PO	90 days	Weaned female calves	Antibody concentration without stimulation	Total immunoglobulin B > A, day 7 & 90	(30)
RCT (N=36, 3)	136 mg/kg DM	A. 0 mg/d Zn proteinate or Zn Met B. 522.88 mg/d Zn proteinate [80 mg/d Zn] C. 467.88 mg/d Zn Met [80 mg/d Zn]	PO	28 days	Holstein calves	Antibody concentration without stimulation	IgG B & C > A IgM C > B > A IgA No difference	(27)
RCT (N=24, 3)	83.9 mg/kg DM	A. 0 mg/d Zn proteinate or Zn oxide B. 627mg/d Zn proteinate [80 mg/d Zn] C. 101mg/d Zn oxide [80 mg/d Zn]	PO	28 days	Newborn calves	Antibody concentration without stimulation	IgG C & B > A, day 14 & 28 IgM C & B > A, day 14 B > A, day 28 IgA No difference	(31)
RCT (N=20, 4)	Not reported	A. 0 mg/kg Zn sulfate & 0 mg/kg Cu sulfate B. 100 mg/kg Zn sulfate [40 mg/kg Zn] C. 25 mg/kg Cu sulfate [9.95 mg/kg Cu] D. 100 mg/kg Zn sulfate & 25 mg/kg Cu sulfate [40 mg/kg Zn, 9.95 mg/kg Cu]	PO	75 days	6 day old crossbred calves	Antibody concentration after immunization against sheep red blood cells *in vitro*	IgG D & B > A & C, day 45 & 75 IgM D & B > A & C, day 45 & 75	(32)
RCT (N=24, 3)	4 mg/kg DM, Zn sulfate	A. 40 mg/kg Zn AA [4.8 mg/kg Zn] B. 80 mg/kg Zn AA [9.6 mg/kg Zn] C. 120 mg/kg Zn AA [14.4 mg/kg Zn]	PO	60 days	1 month old, 69.34 ± 7.67 kg Angus calves	Antibody concentration without stimulation	IgG No difference IgA No difference IgM A & B > C	(33)
RCT (N=90, 3)	26.4 mg/kg DM	A. 0 mg/kg DM Zn Met or Zn oxide B. 25 mg/kg DM Zn Met [5.26 mg/kg Zn] C. 25 mg/kg DM Zn oxide [20 mg/kg Zn]	PO	28 days	214 kg Angus x Hereford steers	Antibody concentration after immunization against BHV and PI3	BHV B & C > A, day 14 PI3 No difference	(34)
RCT (N^1 =^ 84, 3, N^2 =^ 75, 3)	21 mg/kg DM^1^ 38 mg/kg DM^2^	A. 0 mg/d Zn sulfate or Zn AA B. Zn sulfate [360 mg/d Zn] C. Zn as an AA [360 mg/d Zn]	PO	28 days^1^ 140 days^2^	240 ± 1.4 kg Crossbred bulls^1^ 176 ± 2.5 kg beef heifers^2^	Antibody concentration after immunization against BVDV, BRSV, and BHV	BHV^1^ No difference BVDV^1^ B & C > A, day 14 BRSV^1,2^ C > B, day 28^1^ & 56^2^	(43)
RCT (N=60, 4)	Growing phase: 33 mg/kg DM Finishing phase: 26 mg/kg DM	A. 0 mg/kg DM Zn oxide or Zn Proteinate B. 25 mg/kg DM Zn oxide [20.1 mg/kg Zn] C. 25 mg/kg Zn Proteinate [2.5 mg/kg DM Zn] D. 25 mg/kg Zn Proteinate [3.75 mg/kg DM Zn]	PO	Growing phase: 84 days Finishing phase: 84 days or 112 days Total: 168 days or 196 days	246 ± 2 kg Angus & Angus x Hereford steers	Antibody concentration after immunization against BVH	BHV No difference	(28)
RCT (N=24, 4)	44.1 mg/kg DM	A. 0 mg/kg DM Zn sulfate, Zn Met, or Zn Proteinate B. 75 mg/kg DM Zn sulfate [30mg/kg Zn] C. 75 mg/kg DM Zn Met [15.8 mg/kg Zn] D. 75 mg/kg DM Zn propionate [23.2 mg/kg Zn]	PO	21 days	291.1 ± 9.5 kg Crossbred heifers	Antibody concentration after immunization against ovalbumin	No difference	(35)

RCT, randomized controlled trial; Zn, zinc; mg, milligram; kg, kilogram; d, day; DM, dry matter; Met, methionine; Lys, lysine; AA, amino acid complex; PO, per oral; BHV, bovine herpes virus; BVDV, bovine viral diarrhea virus; BRSV, bovine respiratory syncytial virus; PI3, parainfluenza 3 virus; Ig, immunoglobulin. *Only stimulations and outcomes relevant to the assessment of B-cells are listed. ^1^Denotes information relevant to the first experiment in this study. ^2^Denotes information relevant to the second experiment in this study.

Antigen-specific antibody production is a hallmark of vaccination and is essential for controlling disease (45). Zinc supplementation (25 mg/kg DM) tended to increase BHV titers in response to vaccination (34). However, Zn supplementation did not increase BHV titers regardless of the amount and formulation in vaccinated beef calves (28, 43). Zinc (25 mg Zn/kg DM) failed to increase parainfluenza titers in weaned steers (34). Conversely, Zn as an amino acid complex (360 mg/day) increased bovine viral diarrhea virus and bovine respiratory syncytial virus titers compared to Zn sulfate (360 mg/day) in vaccinated beef heifers (43). Finally, Zn supplementation had mixed results when calves were immunized with non-bovine-specific antigens. Non-bovine-specific antigens were only increased in the study where the authors demonstrated an increase in systemic Zn status, making it difficult to interpret the absence of changes in titers at lower Zn doses (32, 35).

Heterogeneity of the dose, formulation, age at supplementation, and vaccines administered limited the ability to compare results among studies. In general, most articles documented higher Ig production in Zn-supplemented animals. However, there were mixed results regarding which outcomes were most affected. The strongest results were for total Ig and non-specific Ig classes, with unanimous improvement of at least one outcome (27, 29–33). Results indicate the possibility of a threshold for the beneficial effects of Zn supplementation. While 32 mg/kg DM - 100 mg/kg DM Zn doses increased Ig production (27–35), higher doses (120 mg/kg DM - 360 mg/day) showed mixed results with no improvement in any of the Ig outcomes in one study (33) and no changes in anti-BHV titers in another (43). Determining the optimal dose, formulation, and age at administration of Zn that has the potential to alter Ig production is an important knowledge gap that warrants further investigation.

### 4.3 Lymphocytes

#### 4.3.1 Lymphocytosis

Zinc supplementation was associated with temporary increases in lymphocyte numbers (**Table 9**) (15, 16). Duration of the lymphocytosis differed between the two studies; however, variations in Zn dose, route of administration, formulation, and concurrent disease may have contributed to the inconsistency. Zinc supplementation (1 mg/kg body weight, subcutaneous Zn edetate) increased circulating lymphocytes for 20 days in 50-day-old calves around weaning (15). However, the lack of immune stimulation in calves limited conclusions about Zn-driven changes in lymphocyte production, as unstimulated lymphocytes in circulation provide limited evidence about the capacity of immune cells to mount an effective response. Moreover, beef heifers supplemented with Zn sulfate (100 mg/kg DM) for 30 days before a challenge with BRD pathogens demonstrated increased lymphocyte counts for 7 days compared to the Zn sulfate (200 mg/kg DM) and Zn Met – Zn sulfate (80 mg/kg DM – 20 mg/kg DM) treatments (16). Nevertheless, the lack of an unsupplemented group in the study limited conclusions about the ability of Zn to increase lymphocyte numbers during disease. Lymphocyte counts alone provide little evidence about changes in overall immune function; future work integrating changes in circulating lymphocyte numbers with functional outcomes would provide a more comprehensive analysis of immune function.

**Table 9:** Summarized results of the peripheral blood mononuclear cell outcomes.

Type of study (N, # groups)	Basal diet Zn	Zn supplement & formulation [Zn dose]	Route	Duration of supplementation	Animals	Outcomes*	Findings	Reference
RCT (N=40, 4)	60 mg/kg	A. 0 mg/kg Zn Met & Zn Lys B. 150 mg/kg Zn Met & Zn Lys [15 mg/kg Zn] C. 300 mg/kg Zn Met & Zn Lys [30 mg/kg Zn] D. 300 mg/kg Zn oxide [168 mg/kg Zn]	PO	6 weeks	6-8 week old Holstein heifer calves	Cytokine concentrations with ex vivo concanavalin A stimulation Blastogenesis in response to ex vivo mitogen stimulation	IL-2 No difference Blastogenesis No difference	(14)
RCT (N=24, 4)	Not reported	A. 0 mg/kg BW Zn edetate & 0 mg/kg Diphenyl diselenide B. 1 mg/kg BW Zn edetate [0.15 mg/kg Zn] C. 0.3 mg/kg BW Diphenyl diselenide [0 mg/kg Zn] D. 1 mg/kg BW Zn edetate & 0.3 mg/kg BW Diphenyl diselenide [0.15 mg/kg Zn]	s.c.	The study lasted 300 days. Supplementation occurred twice on study day 1 and 20.	50 day old male Holstein calves	Circulating numbers without stimulation evaluated on study days 1, 20, and 40	Lymphocyte numbers B > C & A, day 20 B > C & D, day 40	(15)
RCT (N=32, 3)	Not reported	A. 100 mg/kg DM Zn sulphate [40.5 mg/kg Zn] B. 200 mg/kg DM Zn sulphate [81 mg/kg Zn] C. 80 mg/kg DM Zn Met & 20 mg/kg DM Zn sulphate [24.9 mg/kg Zn]	PO	30 days	255 ± 15kg beef heifers	Calves were challenged with BRD pathogens Circulating numbers Cytokines concentrations	Lymphocyte numbers A > B & C IL-6 C > A & B, for 6 hours B < A & C, 6-36 hours IFN γ B > A & C, for 24 hours	(16)
RCT (N=24, 4)	Calf Starter: 46.39 mg/kg DM Wheat straw: 4.29 mg/kg DM Berseem fodder: 19.28 mg/kg DM Milk: 3491 g/L	A. 0 mg/kg DM Zn or Cu nanoparticles B. 32 mg/kg Zn nanoparticles [25.6 mg/kg DM Zn] C. 10mg/kg Cu nanoparticles [0 mg/kg DM Zn] D. 32 mg/kg Zn nanoparticles & 10mg/kg Cu nanoparticles [25.6 mg/kg Zn]	PO	14 days	1 month old dairy calves	Cytokine concentrations without stimulation	TNF D & B > A IFN γ No difference	(29)
RCT (N=36, 3)	136 mg/kg DM	A. 0 mg/d Zn proteinate or Zn Met B. 522.88 mg/d Zn proteinate [80 mg/d Zn] C. 467.88 mg/d Zn Met [80 mg/d Zn]	PO	28 days	Holstein calves	Cytokine concentrations without stimulation	IL-6 No difference IL-8 No difference TGF β No difference IFN γ No difference IL-1 β B & C < A	(27)
RCT (N=24, 3)	83.9 mg/kg DM	A. 0 mg/d Zn proteinate or Zn oxide B. 627 mg/d Zn proteinate [80 mg/d Zn] C. 101 mg/d Zn oxide [80 mg/d Zn]	PO	28 days	Newborn calves	Cytokine concentrations without stimulation	IL-10 C < A, day 14 IL-1 β C < A, day 28 IFN γ No difference	(31)
RCT (N=24, 3)	4 mg/kg DM Zn sulfate	A. 40 mg/kg Zn AA [4.8 mg/kg Zn] B. 80 mg/kg Zn AA [9.6 mg/kg Zn] C. 120 mg/kg Zn AA [14.4 mg/kg Zn]	PO	60 days	1 month old, 69.34 ± 7.67 kg Angus calves	Cytokine concentrations without stimulation	IL-2 B > A > C	(33)
RCT (N=60, 4)	Growing phase: 33 mg/kg DM Zn Finishing phase: 26 mg/kg DM	A. 0 mg/kg DM Zn oxide or Zn proteinate B. 25 mg/kg DM Zn oxide [20.1 mg/kg DM Zn] C. 25 mg/kg Zn proteinate [2.5 mg/kg DM Zn] D. 25 mg/kg Zn proteinate [3.75 mg/kg DM Zn]	PO	Growing phase: 84 days Finishing phase: 84 days or 112 days Total: 168 days or 196 days	246 ± 2 kg Angus & Angus x Hereford steers	Blastogenesis in response to ex vivo mitogen stimulation using phytohemagglutinin and pokeweed mitogen	No difference	(28)

RCT, randomized controlled trial; Zn, zinc; mg, milligram; kg, kilogram; d, day; DM, dry matter; Met, methionine; Lys, lysine; AA, amino acid complex; PO, per oral; s.c., subcutaneous; BRD, bovine respiratory complex; SOD, superoxide dismutase, IFN γ , interferon gamma; TGF β , transforming growth factor beta; IL, interleukin. *Only stimulations and outcomes relevant to the assessment of peripheral blood mononuclear cells are listed.

#### 4.3.2 Cytokine production

Cytokines are proteins secreted by immune cells to induce a response in other immune cells, which is an essential form of intercellular communication for directing immune responses and inflammation (46). Currently, there needs to be more consensus regarding the impact of Zn on cytokine production. Several articles evaluated circulating cytokines and cytokine production as outcomes (**Table 9**), but the marked variation in the cytokine profiles assessed by each study leaves insufficient evidence to determine the extent to which Zn reliably impacts cytokine production. Zinc supplementation (80 mg/kg DM, Zn proteinate, and Zn Met) did not influence plasma IL-8 or TGF- β concentrations in preweaning calves (27). However, Zn supplementation (80 mg/kg DM, Zn oxide) increased serum IL-10 concentrations after 14 days in newborn dairy calves, but this effect was temporary and disappeared by 28 days (31). Additionally, Zn supplementation increased IL-2 concentrations with 80 mg/kg DM Zn as an amino acid complex (33), but not 40 mg/kg DM (33) or doses greater than 80 mg/kg DM (i.e., 120 mg/kg DM - 300 mg/kg DM) in preweaning calves (14, 33). There were mixed results for circulating interferon γ (IFN γ ) concentrations when Zn was supplemented: no change in IFN γ concentrations was reported when 32 to 100 mg/kg DM Zn was supplemented regardless of the Zn formulation (i.e., Zn Met, Zn proteinate, Zn oxide, Zn sulfate, or Zn nanoparticles) (27, 29, 31). However, when Zn sulfate (200 mg/kg DM) was supplemented to beef heifers, IFN γ concentrations after being challenged with BHV and *M. haemolytica* were higher than in groups who received less Zn supplementation (16). Zn supplementation only increased IFN γ concentrations with higher doses of Zn sulfate (200 mg/kg DM) in acute BRD (16). The authors suggested that this was the result of increased pathogen burden, as heifers also had increased vaginal temperature and increased platelet counts (16). In the context of BRD, however, acute inflammation is essential for controlling the disease state, and it is only when that acute inflammation is uncontrolled that it is considered detrimental (47). Given that the IFN γ concentrations were no longer elevated 24 hours after the challenge and every animal received the same challenge dose, it remains unclear if the increased IFN γ concentrations resulted from a robust acute immune response or uncontrolled inflammation.

In general, endogenous pyrogens (i.e., IL-6, tumor necrosing factor, IL-1 β ) demonstrated mixed results but tended to decrease with Zn supplementation (16, 27, 29, 31). When beef heifers were supplemented for 30 days before and during a challenge that modeled BRD, Zn (80 mg/kg DM – 20 mg/kg DM, Zn Met – Zn sulfate) increased IL-6 concentrations for the first 6 hours post-challenge; then, between hours 6 – 36, Zn (200 mg/kg DM, Zn sulfate) decreased IL-6 concentrations (16). However, when preweaning calves were supplemented with organic Zn sources (80 mg/kg DM) without concurrent disease, there were no differences in IL-6 concentrations (27). As a potent proinflammatory cytokine, IL-6 is essential for driving disease-associated inflammation (48). In the context of BRD, an acute increase in IL-6 may be beneficial for controlling infection, while a rapid decline in IL-6 might signal disease resolution and the prevention of uncontrolled inflammation. Moreover, tumor necrosing factor concentrations were reduced by supplementing Zn nanoparticles (32 mg/kg DM) in preweaning calves without concurrent disease (29). Furthermore, Zn supplementation with 80 mg/kg DM, regardless of formulation, decreased circulating IL-1 β concentrations in calves during the first month of life (27, 31). The duration of Zn supplementation was important for IL-1 β concentrations as exhibited by longer (28 days vs. 14 days) supplementation being necessary for IL-1 β decreases (31). Beyond the role proinflammatory cytokines play in controlling the immune response, there is evidence in cattle that tumor necrosing factor and IL-1 β drive inflammation-associated hypophagia (49). In applied work Zn supplemented heifers with BRD had increased DM intake, further supporting Zn as a tool for reducing inflammation-associated hypophagia (50).

The diversity of the cytokine profiles evaluated limited comparisons among studies, but it seems that Zn supplementation reduces proinflammatory cytokine production. Future research is warranted to determine if reductions in proinflammatory cytokine production translate into improved health outcomes for calves. Moreover, differences were observed based on the presence or absence of immune cell stimulation, such as concurrent disease, limiting the comparability of results among studies. Ultimately, there is insufficient evidence to determine the impact of Zn on cytokine production in dairy calves.

#### 4.3.3 Blastogenic responses

Blastogenesis is the process of cells expanding and dividing. The capacity to rapidly expand cell populations is essential for a robust immune response (51). Zinc supplementation did not impact blastogenic responses to mitogens (**Table 9**) (14, 28). It is important to recognize that neither article showed increased serum Zn concentrations with Zn supplementation. However, changes in systemic Zn concentrations were highly dependent on the formulation and dose of Zn supplemented. Similarly, liver Zn concentrations were only increased when Zn Met-Zn Lys was supplemented at 300 mg/kg DM but not at 150 mg/kg DM or when Zn oxide was supplemented (14). Although serum Zn concentrations are of limited use when quantifying an animal’s Zn status, except in the case of overt deficiency (52), it is difficult to interpret a lack of change in lymphocyte function without evidence that Zn supplementation was able to alter systemic Zn status successfully. Moreover, neither article justified the Zn concentrations utilized for improving lymphocyte function, and the dose of Zn necessary to alter lymphocyte functions may differ from that which is required to elevate systemic Zn status. Together, these limitations make it difficult to determine if the lack of change in lymphocyte functions is due to an inappropriate Zn dose or a lack of response to Zn supplementation.

### 4.4 Oxidative stress

Oxidative stress, the cell damage that results from redox imbalance, is known to reduce immune cell functions such as cytokine production, antigen-specific antibody production (53), and proliferation (54). Only one study evaluated oxidative status using reactive oxygen species (ROS) in serum and found that ROS was lower in supplemented animals (15). Malondialdehyde (MDA), a byproduct of lipid peroxidation, was reduced with Zn supplementation in preweaning calves (15, 27, 29, 31, 33). The reduction in MDA was limited to cattle treated with 80 mg/kg DM organic Zn or less, with cattle fed below or above this range not exhibiting changes in MDA (27, 31, 33). Moreover, the formulation of Zn may be important as inorganic Zn at the same dose (80 mg/kg DM) did not reduce MDA (31). Although organic and inorganic Zn formulations are proposed to have similar bioavailability in ruminants, it is suggested that they are metabolized differently (55). This could explain why no difference was observed in serum Zn concentrations between organic and inorganic Zn supplementation despite differences in the OS outcomes (31). Generally, total antioxidant capacity increased in response to Zn supplementation (27, 29, 31, 33). As noted for MDA, the improvement was limited to cattle treated with 80 mg/kg DM Zn or less (27, 29, 33). However, Zn supplemented as Zn proteinate (27) or at 120 mg/kg DM Zn amino acid complex did not improve total antioxidant capacity (33). Zn supplementation had mixed results for antioxidant enzymes with increased activity of glutathione peroxidase (15, 29) and superoxide dismutase activity when supplemented for 60 days or longer (30, 33). However, when Zn was supplemented for less than 60 days, there was no improvement in superoxide dismutase regardless of formulation or dose (15, 27, 29, 31). Glutathione peroxidase was not different when supplemented with Zn as an amino acid complex (33). Moreover, when Zn as an amino acid complex was supplemented at 1,000 mg/day, there was no difference in superoxide dismutase activity within red blood cell lysates from weaned beef steers (17). Furthermore, Zn supplementation did not affect catalase activity (29). This suggests that antioxidant enzyme responsiveness to Zn supplementation may depend on the enzyme being evaluated and the dose, duration, and formulation of Zn used.

Zn supplementation consistently reduced markers of OS (**Table 10**). However, future work analyzing changes in pro-oxidant production relative to antioxidant potential would provide a better understanding of changes in both aspects of redox balance (2). Moreover, MDA is an imprecise biomarker for OS as the thiobarbituric acid utilized in MDA colorimetric assays is a non-specific chromogen prone to cross-reaction (56), and most of the MDA measured is generated ex vivo (57). Isoprostanes offer a more stable byproduct of lipid peroxidation, and their quantification via high-performance liquid chromatography is nowadays considered the gold-standard method for assessing lipid peroxidation (58). Additionally, caution should be used when interpreting individual antioxidants and antioxidant enzymes as an assessment of antioxidant capacity as it does not accurately reflect the complexity of the systemic antioxidant system (59). Furthermore, additional work determining the extent to which these changes in oxidative status translate into improvements in cell function is warranted.

**Table 10:** Summarized results of the oxidant status outcomes.

Type of study (N, # groups)	Basal diet Zn	Zn supplement & formulation [Zn dose]	Route	Duration of supplementation	Animals	Outcomes*	Findings	Reference
RCT (N=24, 4)	Not reported	A. 0 mg/kg BW Zn edetate & 0 mg/kg Diphenyl diselenide B. 1 mg/kg BW Zn edetate [0.15 mg/kg Zn] C. 0.3 mg/kg BW Diphenyl diselenide [0 mg/kg Zn] D. 1 mg/kg BW Zn edetate & 0.3 mg/kg BW Diphenyl diselenide [0.15 mg/kg Zn]	s.c.	The study lasted 300 days. Supplementation occurred twice on study day 1 and 20.	50 day old male Holstein calves	ROS MDA GPx SOD	ROS B, C, & D > A, day 20 & 40 MDA D < A, B, & C, day 20 B, C, & D < A, day 40 ↑ GPx C & D > A & B, day 20 B & C > A, day 40 SOD No difference	(15)
RCT (N=48, 2)	0 mg/kg	A. 7 g/d organic trace mineral mix [951 mg/d Zn]	PO	42 days	284 ± 25 kg angus crossbred steers	CuZn SOD Mn SOD Total SOD	CuZn SOD No difference Mn SOD No difference Total SOD No difference	(17)
		B. 7 g/d organic trace mineral mix & 1000 mg/d Zn AA for 14 days then only the organic trace mineral mix [1900, 929 mg/d Zn]		14 days, 28 days				
		C. Inorganic trace mineral mix formulated to match group A. [1095 mg/d Zn]		42 days				
RCT (N=24, 4)	Calf Starter: 46.39 mg/kg DM Wheat straw: 4.29 mg/kg DM Berseem fodder: 19.28 mg/kg DM Milk: 3491 g/L	A. 0 mg/kg DM Zn or Cu nanoparticles B. 32 mg/kg Zn nanoparticles [25.6 mg/kg Zn] C. 10mg/kg Cu nanoparticles [0 mg/kg Zn] D. 32 mg/kg Zn nanoparticles & 10mg/kg Cu nanoparticles [25.6 mg/kg Zn]	PO	14 days	1 month old dairy calves	MDA TAC GPx SOD Catalase	MDA D > A, B, & C TAC D > A, B, & C GPx activity cD > B & C > A SOD D > A, B, & C Catalase No difference	(29)
RCT (N=20, 2)	Calf starter: 30 mg/kg DM Milk replacer: 40 mg/kg DM	A. 0 mg/kg DM Zn Met B. 30 mg/kg DM Zn Met [6.3 mg/kg Zn]	PO	90 days	Weaned female calves	SOD	SOD B > A, day 7 & 90	(30)
RCT (N=36, 3)	136 mg/kg DM	A. 0 mg/d Zn proteinate or Zn Met B. 522.88 mg/d Zn proteinate [80 mg/d Zn] C. 467.88 mg/d Zn Met [80 mg/d Zn]	PO	28 days	Holstein calves	MDA TAC SOD	MDA B & C < A TAC C > A & B SOD No difference	(27)
RCT (N=24, 3)	83.9 mg/kg DM	A. 0 mg/d Zn proteinate or Zn oxide B. 627 mg/d Zn proteinate [80 mg/d Zn] C. 101 mg/d Zn oxide [80 mg/d Zn]	PO	28 days	Newborn calves	MDA TAC SOD	MDA B < C & A, day 14 B & C < A, day 28 TAC B & C > A, day 14 SOD No difference	(31)
RCT (N=24, 3)	4 mg/kg DM Zn sulfate	A. 40 mg/kg Zn AA [4.8 mg/kg Zn] B. 80 mg/kg Zn AA [9.6 mg/kg Zn] C. 120 mg/kg Zn AA [14.4 mg/kg Zn]	PO	60 days	1 month old, 69.34 ± 7.67 kg Angus calves	MDA TAC GPx SOD	MDA B & A < C TAC A & B > C GPx No difference SOD B > C	(33)

RCT, randomized controlled trial; Zn, zinc, mg=milligram; kg, kilogram; d, day; DM, dry matter; Met, methionine; Lys, lysine; AA, amino acid complex; PO, per oral; s.c., subcutaneous; MDA, malondialdehyde; TAC, total antioxidant capacity; GPx, glutathione peroxidase; SOD, superoxide dismutase. *Only stimulations and outcomes relevant to the assessment of oxidative stress are listed.

## 5 Applications for calves

There is substantial evidence for the role of Zn in immunity. For a more comprehensive discussion of the topic, please refer to the review by Wessels, Maywald (7). Zinc deficiency is associated with immunosuppression and has been fundamental in demonstrating the importance of Zn for immune function (10, 59). This has been well characterized in Holstein dairy calves due to the lethal trait A46, a genetic disorder that compromises intestinal Zn absorption (10, 59). Calves with A46 have marked reductions in circulating lymphocyte numbers and lymphocyte function (10).

The recommendations for Zn supplementation in dairy calves are formulated to prevent nutritional deficiencies (11). However, these industry recommendations do not account for the potential benefits associated with Zn supplementation above these recommendations during periods of stress or disease, as demonstrated in non-bovine species (60–62). Many articles discuss the benefits of supplemental Zn in reducing the incidence of diarrhea (27, 63, 64) and expediting recovery from diarrhea in dairy calves (65). Similarly, Zn may improve calf recovery from respiratory disease as calves challenged with BHV that were supplemented with Zn had increased DM intake and required fewer days to recover post-BHV-challenge than unsupplemented counterparts (50). Future research is warranted to investigate these differences as they could influence Zn administration under field conditions.

## 6 Future directions

Even though substantial evidence supports an essential role for Zn and immunity, much work remains to be done characterizing Zn’s mechanistic role in modulating immune cell function in dairy calves. The human literature supports the role of Zn in maintaining redox balance and mitigating OS (13, 66, 67). For example, Zn reduced OS and improved lymphocyte function in elderly adults (68). In calves, there was evidence that Zn mitigated OS, future work characterizing how reductions in OS translate into improved lymphocyte functionality and calf health is warranted. More recently, immunometabolism has become an emerging field, driven by the recognized influence of metabolites on immune cell signaling and function (69). Work characterizing the role of Zn in supporting immune cell metabolism and mitochondrial function is emerging in model organisms and human literature (70, 71). Future work harnessing metabolic and mitochondrial outputs is an opportunity worth exploring to address the reduced functionality of calf lymphocytes. Finally, while there is evidence to support Zn supplementation to reduce the duration and severity of clinical signs during disease in dairy calves, more mechanistic research is warranted to elucidate the extent to which Zn supports the immune system of dairy calves. Moreover, if Zn proves successful in modulating dairy calf immune function, more work evaluating novel approaches for supplementation will be necessary. For example, some initial work assessing pregnant cow trace mineral supplementation showed some promising results for improving the offspring’s immunity (72), suggesting that immunomodulation could start in the prenatal stage.

## 7 Concluding remarks

This scoping review highlights important knowledge gaps for the role of Zn in dairy calf immunity. There is evidence to support Zn supplementation above nutritional requirements for improving circulating cell numbers, but results are highly variable for each immune cell type. Moreover, there is moderate evidence to support the reduction of proinflammatory cytokines and increased antibody production. Furthermore, there was evidence to support Zn shortening the duration of clinical signs in calves with respiratory and gastrointestinal illness as well as improving DM intake in sick calves. However, there was a lack of research regarding the capacity of Zn to improve immune cell functionality. Furthermore, the heterogeneity in dosage, route, formulation, and duration of Zn administered to calves limited the integration of results among studies. Finally, while there is evidence to support Zn supplementation to calves while facing a disease, more mechanistic research is warranted to elucidate the extent to which Zn supports the immune system of calves.

## Author contributions

KK: Conceptualization, Funding acquisition, Investigation, Methodology, Writing – original draft. AA: Funding acquisition, Supervision, Writing – review & editing.

## References

[1] Overton TR Yasui T. Practical applications of trace minerals for dairy cattle. J Anim Sci. (2014) 92:416–26. doi: 10.2527/jas.2013-7145 24305870

[2] Abuelo A Hernandez J Benedito JL Castillo C. Oxidative stress index (OSi) as a new tool to assess redox status in dairy cattle during the transition period. Animal. (2013) 7:1374–8. doi: 10.1017/S1751731113000396 23510791

[3] Abuelo A Perez-Santos M Hernandez J Castillo C. Effect of colostrum redox balance on the oxidative status of calves during the first 3 months of life and the relationship with passive immune acquisition. Vet J. (2014) 199:295–9. doi: 10.1016/j.tvjl.2013.10.032 24332736

[4] Cuervo W Sordillo LM Abuelo A. Oxidative stress compromises lymphocyte function in neonatal dairy calves. Antioxid (Basel). (2021) 10:255. doi: 10.3390/antiox10020255 PMC7915147 33562350

[5] Nayak A Abuelo A. Parenteral antioxidant supplementation at birth improves the response to intranasal vaccination in newborn dairy calves. Antioxid (Basel). (2021) 10:1979. doi: 10.3390/antiox10121979 PMC8750176 34943082

[6] Carlson H Cullens-Nobis FM Owczarzak EJ Abuelo A. Effect of parenteral micronutrient supplementation at birth on immunity, growth, and health in pre-weaning dairy heifers. J Dairy Sci. (2024). doi: 10.3168/jds.2023-24292 38331183

[7] Wessels I Maywald M Rink L. Zinc as a gatekeeper of immune function. Nutrients. (2017) 9:1286. doi: 10.3390/nu9121286 29186856 PMC5748737

[8] Blackmon DM Miller WJ Morton JD. Zinc deficiency in ruminants. Occurrence, effects, diagnosis and treatments. Vet Med Small Anim Clin. (1967) 62:265–70. 5182718

[9] Shankar AH Prasad AS. Zinc and immune function: the biological basis of altered resistance to infection. Am J Clin Nutr. (1998) 68:447S–63S. doi: 10.1093/ajcn/68.2.447S 9701160

[10] Perryman LE Leach DR Davis WC Mickelsen WD Heller SR Ochs HD. Lymphocyte alterations in zinc-deficient calves with lethal trait A46. Vet Immunol Immunopathol. (1989) 21:239–48. doi: 10.1016/0165-2427(89)90034-2 2800326

[11] NASEM, National Academies of Sciences, Engineering, Medicine. Nutrient Requirements of Dairy Cattle. Washington, DC: The National Academies Press (2021). 502 p 38386771

[12] De K Pal S Mukherjee J Prasad S Dang AK. Effect of *in vitro* copper and zinc supplementation on neutrophil phagocytic activity and lymphocyte proliferation response of transition dairy cows. Agr Res. (2015) 4:388–95. doi: 10.1007/s40003-015-0181-7

[13] Prasad AS Bao B Beck FW Kucuk O Sarkar FH. Antioxidant effect of zinc in humans. Free Radic Biol Med. (2004) 37:1182–90. doi: 10.1016/j.freeradbiomed.2004.07.007 15451058

[14] Kincaid RL Chew BP Cronrath JD. Zinc oxide and amino acids as sources of dietary zinc for calves: effects on uptake and immunity. J Dairy Sci. (1997) 80:1381–8. doi: 10.3168/jds.S0022-0302(97)76067-3 9241600

[15] Santos DSD Boito JP Klauck V Reis JHD Gebert RR Glombowsky P. Health benefits of subcutaneous zinc edetate and diphenyl diselenide in calves during the weaning period. Acad Bras Cienc. (2019) 91:e20171042. doi: 10.1590/0001-3765201920171042 30994751

[16] Broadway PR Carroll J Burdick Sanchez N Word A Roberts S Kaufman E. Zinc source and concentration altered physiological responses of beef heifers during a combined viral-bacterial respiratory challenge. Anim (Basel). (2021) 11(3):646. doi: 10.3390/ani11030646 PMC8000065 33804483

[17] Smerchek DT Branine ME McGill JL Hansen SL. Effects of supplemental Zn concentration and trace mineral source on immune function and associated biomarkers of immune status in weaned beef calves received into a feedlot. J Anim Sci. (2023) 101:1–12. doi: 10.1093/jas/skac428 PMC9910396 36588522

[18] Price J Wood DA. Zinc responsive parakeratosis and ill-thrift in a Friesian calf. Vet Rec. (1982) 110:478. doi: 10.1136/vr.110.20.478 7101710

[19] Legg SP Sears L. Zinc sulphate treatment of parakeratosis in cattle. Nature. (1960) 186:1061–2. doi: 10.1038/1861061a0 14415314

[20] Haaranen S. The effect of zinc on itching tail-root eczema in cattle. Nordisk Veterinaermedicin. (1962) 14:265–9

[21] Rickard BF. Facial eczema: zinc responsiveness in dairy cattle. N Z Vet J. (1975) 23:41–2. doi: 10.1080/00480169.1975.34190 1058361

[22] Bennison JJ Nottingham RM Key EL Parkins JJ. The effect of zinc oxide and elemental zinc boluses on the concentrations of Zn in serum and faeces, and on providing protection from natural Pithomyces chartarum challenge in calves. N Z Vet J. (2010) 58:196–200. doi: 10.1080/00480169.2010.68865 20676157

[23] Miller WJ Blackmon DM Hiers JM Jr. Fowler PR Clifton CM Gentry RP. Effects of adding two forms of supplemental zinc to a practical diet on skin regeneration in Holstein heifers and evaluation of a procedure for determining rate of wound healing. J Dairy Sci. (1967) 50:715–21. doi: 10.3168/jds.S0022-0302(67)87499-X 6038880

[24] Li Y Pan M Meng S Xu W Wang S Dou M. The effects of zinc oxide nanoparticles on antioxidation, inflammation, tight junction integrity, and apoptosis in heat-stressed bovine intestinal epithelial cells *in vitro* . Biol Trace Elem Res. (2023) 202(5):2042–51. doi: 10.1007/s12011-023-03826-6 37648935

[25] Ma FT Wo YQL Shan Q Wei JY Zhao SG Sun P. Zinc-methionine acts as an anti-diarrheal agent by protecting the intestinal epithelial barrier in postnatal Holstein dairy calves. Anim Feed Sci Technol. (2020) 270:114686. doi: 10.1016/j.anifeedsci.2020.114686

[26] Golynski M Lutnicki K Kostro K. Effect of oral administration of zinc sulfate with simultaneous use of nonspecific immunostimulation on the course of trichophytosis in beef cattle. Bull Vet Inst Pulawy. (2012) 56:149–53

[27] Wo Y Jin Y Gao D Ma F Ma Z Liu Z. Supplementation with zinc proteinate increases the growth performance by reducing the incidence of diarrhea and improving the immune function of dairy calves during the first month of life. Front Vet Sci. (2022) 9:911330. doi: 10.3389/fvets.2022.911330 35847636 PMC9284037

[28] Spears JW Kegley EB. Effect of zinc source (zinc oxide vs zinc proteinate) and level on performance, carcass characteristics, and immune response of growing and finishing steers. J Anim Sci. (2002) 80:2747–52. doi: 10.2527/2002.80102747x 12413098

[29] Pandey P Kumar M Kumar V Kushwaha R Vaswani S Kumar A. The dietary supplementation of copper and zinc nanoparticles improves health condition of young dairy calves by reducing the incidence of diarrhoea and boosting immune function and antioxidant activity. Biol Trace Elem Res. (2023) 201:3791–803. doi: 10.1007/s12011-022-03481-3 36370333

[30] Dresler S Illek J Zeman L. Effects of organic zinc supplementation in weaned calves. Acta Vet Brno. (2016) 85:49–54. doi: 10.2754/avb201685010049

[31] Liu J Ma F Degen A Sun P. The effects of zinc supplementation on growth, diarrhea, antioxidant capacity, and immune function in holstein dairy calves. Anim (Basel). (2023) 13(15):2493. doi: 10.3390/ani13152493 PMC10417456 37570301

[32] Prasad T Kundu MS. Serum IgG and IgM responses to sheep red blood cells (SRBC) in weaned calves fed milk supplemented with Zn and Cu. Nutrition. (1995) 11:712–5 8748260

[33] Hou P Li B Wang Y Li D Huang X Sun W. The effect of dietary supplementation with zinc amino acids on immunity, antioxidant capacity, and gut microbiota composition in calves. Anim (Basel). (2023) 13(9):1570. doi: 10.3390/ani13091570 PMC10177098 37174607

[34] Spears JW Harvey RW Brown TT Jr. Effects of zinc methionine and zinc oxide on performance, blood characteristics, and antibody titer response to viral vaccination in stressed feeder calves. J Am Vet Med Assoc. (1991) 199:1731–3. doi: 10.2460/javma.1991.199.12.1731 1667527

[35] Nunnery GA Vasconcelos JT Parsons CH Salyer GB Defoor PJ Valdez FR. Effects of source of supplemental zinc on performance and humoral immunity in beef heifers. J Anim Sci. (2007) 85:2304–13. doi: 10.2527/jas.2007-0167 17526672

[36] Chvapil M Stankova L Zukoski Ct Zukoski C 3rd. Inhibition of some functions of polymorphonuclear leukocytes by *in vitro* zinc. J Lab Clin Med. (1977) 89:135–46. 830774

[37] Bancroft B. Immunology simplified. Semin Perioper Nurs. (1994) 3:70–8. 7894425

[38] El-Maghraby MM Mahmoud AE. Clinical, hematological, and biochemical studies on hypozincemia in neonatal calves in Egypt. Vet World. (2021) 14:314–8. doi: 10.14202/vetworld. PMC7994119 33776296

[39] Miller WJ Morton JD Pitts WJ Clifton CM. Effect of zinc deficiency and restricted feeding on wound healing in the bovine. Proc Soc Exp Biol Med. (1965) 118:427–30. doi: 10.3181/00379727-118-29865 14268643

[40] Kolanus W Romeo C Seed B. T cell activation by clustered tyrosine kinases. Cell. (1993) 74:171–83. doi: 10.1016/0092-8674(93)90304-9 8334702

[41] Shipkova M Wieland E. Surface markers of lymphocyte activation and markers of cell proliferation. Clin Chim Acta. (2012) 413:1338–49. doi: 10.1016/j.cca.2011.11.006 22120733

[42] Bjørneboe M Gormsen H. Experimental studies on the role of plasma cells as antibody producers. 1942. Apmis. (2007) 115:440–83; discussion 84-5. doi: 10.1111/j.1600-0463.2007.apm_681a.x 17504402

[43] Kegley EB Silzell SA Kreider DL Galloway DL Coffey KP Hornsby JA. The Immune Response and Performance of Calves Supplemented with Zinc from an Organic and an Inorganic Source11Published with the approval of the director of the Arkansas Agricultural Experiment Station, manuscript no. 00075. Prof Anim Sci. (2001) 17:33–8. doi: 10.15232/S1080-7446(15)31593-X

[44] Mach JP Pahud JJ. Secretory IgA, a major immunoglobulin in most bovine external secretions. J Immunol. (1971) 106:552–63. doi: 10.4049/jimmunol.106.2.552 4100470

[45] Osburn BI Stott JL. Immune response to vaccination. Adv Vet Sci Comp Med. (1989) 33:93–108. doi: 10.1016/B978-0-12-039233-9.50007-X 2648778

[46] Borish LC Steinke JW. 2. Cytokines and chemokines. J Allergy Clin Immunol. (2003) 111:S460–75. doi: 10.1067/mai.2003.108 12592293

[47] Levy BD Serhan CN. Resolution of acute inflammation in the lung. Annu Rev Physiol. (2014) 76:467–92. doi: 10.1146/annurev-physiol-021113-170408 PMC4295924 24313723

[48] Kopf M Baumann H Freer G Freudenberg M Lamers M Kishimoto T. Impaired immune and acute-phase responses in interleukin-6-deficient mice. Nature. (1994) 368:339–42. doi: 10.1038/368339a0 8127368

[49] Godson DL Baca-Estrada ME Van Kessel AG Hughes HP Morsy MA Van Donkersgoed J. Regulation of bovine acute phase responses by recombinant interleukin-1 beta. Can J Vet Res. (1995) 59:249–55. PMC1263778 8548685

[50] Chirase NK Hutcheson DP Thompson GB. Feed intake, rectal temperature, and serum mineral concentrations of feedlot cattle fed zinc oxide or zinc methionine and challenged with infectious bovine rhinotracheitis virus. J Anim Sci. (1991) 69:4137–45. doi: 10.2527/1991.69104137x 1778828

[51] Colley DG DeWitt CW. Mixed lymphocyte blastogenesis in response to multiple histocompatibility antigens. J Immunol. (1969) 102:107–16. doi: 10.4049/jimmunol.102.1.107 5765450

[52] Hambidge KM Casey CE Krebs NF. Zinc. Trace Elements in Human and Animal Nutrition. 2. San Diego, CA: Academic Press, Inc (1986) p. 13–22

[53] Cemerski S van Meerwijk JP Romagnoli P. Oxidative-stress-induced T lymphocyte hyporesponsiveness is caused by structural modification rather than proteasomal degradation of crucial TCR signaling molecules. Eur J Immunol. (2003) 33:2178–85. doi: 10.1002/eji.200323898 12884292

[54] Kasic T Colombo P Soldani C Wang CM Miranda E Roncalli M. Modulation of human T-cell functions by reactive nitrogen species. Eur J Immunol. (2011) 41:1843–9. doi: 10.1002/eji.201040868 21480210

[55] Spears JW. Zinc methionine for ruminants: relative bioavailability of zinc in lambs and effects of growth and performance of growing heifers. J Anim Sci. (1989) 67:835–43. doi: 10.2527/jas1989.673835x 2722712

[56] Knight JA Pieper RK McClellan L. Specificity of the thiobarbituric acid reaction: its use in studies of lipid peroxidation. Clin Chem. (1988) 34:2433–8. doi: 10.1093/clinchem/34.12.2433 3197281

[57] Celi P. Biomarkers of oxidative stress in ruminant medicine. Immunopharmacol Immunotoxicol. (2011) 33:233–40. doi: 10.3109/08923973.2010.514917 20849293

[58] Kadiiska MB Gladen BC Baird DD Germolec D Graham LB Parker CE. Biomarkers of oxidative stress study II: are oxidation products of lipids, proteins, and DNA markers of CCl4 poisoning? Free Radic Biol Med. (2005) 38:698–710. doi: 10.1016/j.freeradbiomed.2004.09.017 15721980

[59] Brummerstedt E Andresen E Basse A Flagstad T. Lethal trait A 46 in cattle. Immunological investigations. Nord Vet Med. (1974) 26:279–93. 4208828

[60] Katouli M Melin L Jensen-Waern M Wallgren P Möllby R. The effect of zinc oxide supplementation on the stability of the intestinal flora with special reference to composition of coliforms in weaned pigs. J Appl Microbiol. (1999) 87:564–73. doi: 10.1046/j.1365-2672.1999.00853.x 10583685

[61] Højberg O Canibe N Poulsen HD Hedemann MS Jensen BB. Influence of dietary zinc oxide and copper sulfate on the gastrointestinal ecosystem in newly weaned piglets. Appl Environ Microbiol. (2005) 71:2267–77. doi: 10.1128/AEM.71.5.2267-2277.2005 PMC1087531 15870311

[62] Bhutta ZA Bird SM Black RE Brown KH Gardner JM Hidayat A. Therapeutic effects of oral zinc in acute and persistent diarrhea in children in developing countries: pooled analysis of randomized controlled trials. Am J Clin Nutr. (2000) 72:1516–22. doi: 10.1093/ajcn/72.6.1516 11101480

[63] Glover AD Puschner B Rossow HA Lehenbauer TW Champagne JD Blanchard PC. A double-blind block randomized clinical trial on the effect of zinc as a treatment for diarrhea in neonatal Holstein calves under natural challenge conditions. Prev Vet Med. (2013) 112:338–47. doi: 10.1016/j.prevetmed.2013.09.001 PMC7114245 24074841

[64] Chang MN Wei JY Hao LY Ma FT Li HY Zhao SG. Effects of different types of zinc supplement on the growth, incidence of diarrhea, immune function, and rectal microbiota of newborn dairy calves. J Dairy Sci. (2020) 103:6100–13. doi: 10.3168/jds.2019-17610 32307167

[65] Feldmann HR Williams DR Champagne JD Lehenbauer TW Aly SS. Effectiveness of zinc supplementation on diarrhea and average daily gain in pre-weaned dairy calves: A double-blind, block-randomized, placebo-controlled clinical trial. PloS One. (2019) 14:e0219321. doi: 10.1371/journal.pone.0219321 31291305 PMC6619766

[66] Prasad AS. Zinc is an antioxidant and anti-inflammatory agent: its role in human health. Front Nutr. (2014) 1:14. doi: 10.3389/fnut.2014.00014 25988117 PMC4429650

[67] Kloubert V Rink L. Zinc as a micronutrient and its preventive role of oxidative damage in cells. Food Funct. (2015) 6:3195–204. doi: 10.1039/C5FO00630A 26286461

[68] Prasad AS Beck FW Bao B Fitzgerald JT Snell DC Steinberg JD. Zinc supplementation decreases incidence of infections in the elderly: effect of zinc on generation of cytokines and oxidative stress. Am J Clin Nutr. (2007) 85:837–44. doi: 10.1093/ajcn/85.3.837 17344507

[69] O’Neill LA Kishton RJ Rathmell J. A guide to immunometabolism for immunologists. Nat Rev Immunol. (2016) 16:553–65. doi: 10.1038/nri.2016.70 PMC5001910 27396447

[70] Peng-Winkler Y Wessels I Rink L Fischer HJ. Zinc levels affect the metabolic switch of T cells by modulating glucose uptake and insulin receptor signaling. Mol Nutr Food Res. (2022) 66:e2100944. doi: 10.1002/mnfr.202100944 35182109

[71] Zhang G Sheng M Wang J Teng T Sun Y Yang Q. Zinc improves mitochondrial respiratory function and prevents mitochondrial ROS generation at reperfusion by phosphorylating STAT3 at Ser(727). J Mol Cell Cardiol. (2018) 118:169–82. doi: 10.1016/j.yjmcc.2018.03.019 29605530

[72] Jacometo CB Osorio JS Socha M Corrêa MN Piccioli-Cappelli F Trevisi E. Maternal consumption of organic trace minerals alters calf systemic and neutrophil mRNA and microRNA indicators of inflammation and oxidative stress. J Dairy Sci. (2015) 98:7717–29. doi: 10.3168/jds.2015-9359 26319761

